# Biomass Enzymatic Saccharification Is Determined by the Non-KOH-Extractable Wall Polymer Features That Predominately Affect Cellulose Crystallinity in Corn

**DOI:** 10.1371/journal.pone.0108449

**Published:** 2014-09-24

**Authors:** Jun Jia, Bin Yu, Leiming Wu, Hongwu Wang, Zhiliang Wu, Ming Li, Pengyan Huang, Shengqiu Feng, Peng Chen, Yonglian Zheng, Liangcai Peng

**Affiliations:** 1 National Key Laboratory of Crop Genetic Improvement and National Centre of Plant Gene Research (Wuhan), Huazhong Agricultural University, Wuhan, P.R. China; 2 Biomass and Bioenergy Research Centre, Huazhong Agricultural University, Wuhan, P.R. China; 3 College of Plant Science and Technology, Huazhong Agricultural University, Wuhan, P.R. China; 4 College of Life Science and Technology, Huazhong Agricultural University, Wuhan, P.R. China; 5 Institute of Crop Sciences, Chinese Academy of Agricultural Sciences, Beijing, P.R. China; University of Georgia, United States of America

## Abstract

Corn is a major food crop with enormous biomass residues for biofuel production. Due to cell wall recalcitrance, it becomes essential to identify the key factors of lignocellulose on biomass saccharification. In this study, we examined total 40 corn accessions that displayed a diverse cell wall composition. Correlation analysis showed that cellulose and lignin levels negatively affected biomass digestibility after NaOH pretreatments at *p*<0.05 & 0.01, but hemicelluloses did not show any significant impact on hexoses yields. Comparative analysis of five standard pairs of corn samples indicated that cellulose and lignin should not be the major factors on biomass saccharification after pretreatments with NaOH and H_2_SO_4_ at three concentrations. Notably, despite that the non-KOH-extractable residues covered 12%–23% hemicelluloses and lignin of total biomass, their wall polymer features exhibited the predominant effects on biomass enzymatic hydrolysis including Ara substitution degree of xylan (reverse Xyl/Ara) and S/G ratio of lignin. Furthermore, the non-KOH-extractable polymer features could significantly affect lignocellulose crystallinity at *p*<0.05, leading to a high biomass digestibility. Hence, this study could suggest an optimal approach for genetic modification of plant cell walls in bioenergy corn.

## Introduction

Lignocellulose has been regarded as a major biomass resource for biofuels and chemicals [1], [2]. Traditional field crops constitute the bulk of lignocellulosic resources, and thus the application of such materials complements that of food supplies. Lignocellulosic biomass process involves three major steps: physical and chemical pretreatments to disrupt the cell wall; enzymatic hydrolysis to release soluble sugar; and yeast fermentation to produce ethanol. However, plant cell wall recalcitrance entails a costly biomass process to produce biofuels [3], [4]. Principally, recalcitrance is characterized by cell wall composition and wall polymer features [5]–[8]. To reduce recalcitrance, genetic modification of plant cell walls has been proposed as a promising solution in bioenergy crops [9]–[12]. Hence, the key factors of plant cell walls affecting biomass enzymatic saccharification should be identified in various pretreatment conditions.

Plant cell walls are mainly composed of cellulose, hemicelluloses, and lignin. Cellulose is a crystalline linear polymer of β-(1,4)-linked glucose moieties, accounting for approximately 30% of the dry mass of primary cell walls and a maximum of 40% of secondary cell walls [13], [14]. It has been characterized that cellulose crystallinity is the key factor that negatively affects biomass enzymatic digestions in plants [5], [7], [15].

Hemicelluloses are the polysaccharides accounting for approximately 20% to 35% of lignocellulosic biomass [16]. Hemicelluloses can be effectively extracted using different concentrations of alkali that dissociates the hydrogen bonds of wall polymers [17]. For example, 4 M KOH has been used to remove hemicelluloses and other associated wall polymers in plants [6], [8], [17]. Hemicelluloses have been considered as the positive factor affecting biomass digestibility in *Miscanthus* [5], but they are not the main factors on biomass digestibility in rice, wheat and sweet sorghum [8], [33]. Although xylan is the major hemicellulose in grasses, the degree of arabinose (Ara) substitution is reported as the main factor positively affecting biomass enzymatic saccharification in plants [6], [8], [33].

Lignin is a very stable phenolic polymer composed of *p*-coumaryl alcohol (H), coniferyl alcohol (G), and sinapyl alcohol (S). Lignin has been considered as the major contributor to lignocellulosic recalcitrance because of its structural diversity and heterogeneity. However, recent reports have indicated that lignin could play dual roles in biomass enzymatic digestions due to the distinctive lignin compositions and monolignol ratios in different plant species [5], [8], [18], [19], [34].

As a highly photosynthetic-efficient C4 grass, corn is one of the major food crops with large amounts of lignocellulosic residues that can be used for biofuels [20]. Despite various pretreatment technologies have been applied in corn lignocellulose digestions [21]–[26], limited information is available regarding cell wall characteristics that affect biomass digestibility in corn. However, due to the complicated structures and diverse biological functions of plant cell walls, it becomes technically difficult to identify the main factors on biomass digestions. In the present study, we determined total 40 natural corn accessions that displayed a diverse cell wall composition. We then selected five standard pairs of corn samples that exhibited characteristic cell wall composition and features. Hence, the current study focused on identification of the main factors of the three major wall polymers that affect biomass enzymatic digestibility under various chemical pretreatments in corn stalk.

## Materials and Methods

### Plant materials

Total 539 corn accessions collected from China, American and International Maize and Wheat Improvement Center (CIMMYT) were planted in Jianshui, Yunnan, China in 2010. Among them, 40 accessions were used in this study. Five matured plants of each accession were harvested, and the stem tissues without leaves were dried at 50°C. The dried tissues were ground through a 40 mesh sieve and stored in a dry container until use. In addition, the field study was carried out on private exprimental land and did not involve endangered or protected species, and no specific permissions were required.

### Plant cell wall fractionation

The plant cell wall fractionation method was described by Peng et al. [17] and Xu et al. [5] with minor modification. The well-mixed biomass powder samples were used for cell wall fractionation. The soluble sugar, lipids, starch and pectin of the samples were consecutively removed by potassium phosphate buffer (pH 7.0), chloroform-methanol (1∶1, v/v), DMSO-water (9∶1, v/v) and 0.5% (w/v) ammonium oxalate. The remaining pellet was extracted with 4 M KOH with 1.0 mg/mL sodium borohydride for 1 h at 25°C, and the combined supernatant with two parallels, one parallel was neutralized, dialyzed and lyophilized as KOH-extractable hemicelluloses monosaccharides; and one parallel was collected for determination of free pentoses and hexoses as the KOH-extractable hemicelluloses. The non-KOH-extractable residues were sequentially extracted with trifluoroacetic acid (TFA) at 120°C for 1 h as non-KOH-extractable hemicelluloses, and the remaining pellet was used as crystalline cellulose. All experiments were performed in independent triplicate.

### Colorimetric assay of hexoses and pentoses

UV-VIS Spectrometer (V-1100D, Shanghai MAPADA Instruments Co., Ltd. Shanghai, China) was used for total hexoses and pentoses assay as described by Huang et al. [27] and Wu et al. [8]. Hexoses were detected using the anthrone/H_2_SO_4_ method [14] and pentoses were tested using the orcinol/HCl method [28]. Anthrone was purchased from Sigma-Aldrich Co. LLC., and ferric chloride and orcinol were obtained from Sinopharm Chemical Reagent Co., Ltd. The standard curves for hexoses and pentoses were drawn using D-glucose and D-xylose as standards (purchased from Sinopharm Chemical Reagent Co., Ltd.) respectively. Total sugar yield from pretreatment and enzymatic hydrolysis was subject to the sum total of hexoses and pentoses. Considering the high pentoses level can affect the absorbance reading at 620 nm for hexoses content by anthrone/H_2_SO_4_ method, the deduction from pentoses reading at 660 nm was carried out for final hexoses calculation. A series of xylose concentrations were analyzed for plotting the standard curve referred for the deduction, which was verified by gas chromatography-mass spectrometry (GC-MS) analysis. All experiments were carried out in triplicate.

### GC-MS determination of hemicelluloses monosaccharides

Determination of monosaccharide composition of hemicelluloses by GC-MS was described by Li et al. [6] and Wu et al. [8] with minor modification. The combined supernatants from 4 M KOH fraction were dialyzed for 36 h after neutralization with acetic acid. The sample from the dialyzed KOH-extractable supernatant or the non-KOH-extractable residue was hydrolyzed by 2 M TFA for free monosaccharide release in a sealed tube at 121°C in an autoclave for 1 h. *Myo*-inositol (200 µg) was added as the internal standard for GC-MS (SHIMADZU GCMS-QP2010 Plus) analysis.

GC-MS analytical conditions: Restek Rxi-5 ms, 30 m×0.25 mm ID×0.25 µm df column; carrier gas: helium; injection method: split; injection port: 250°C; interface: 250°C; injection volume: 1.0 µL; the temperature program: from 155°C (held for 23 min) to 200°C (held for 5 min) at 3.8°C/min, and then from 200°C to 300°C (held for 2 min) at 20°C/min; ion source temperature: 200°C; ACQ Mode: SIM. The mass spectrometer was operated in the EI mode with ionization energy of 70 ev. Mass spectra were acquired with full scans based on the temperature program from 50 to 500 m/z in 0.45 s. Calibration curves of all analytes routinely yielded correlation coefficients of 0.999 or better.

### Total lignin measurement and high performance liquid chromatography (HPLC) detection of lignin monomers

Total lignin was determined by two-step acid hydrolysis method according to Laboratory Analytical Procedure of the National Renewable Energy Laboratory [29], as described by Wu et al. [8]. All samples were carried out in triplicate.

Lignin monomer determination was described by Wu et al. [8]. Standard chemicals: *p*-Hydroxybenzaldehyde (H), vanillin (G) and syringaldehyde (S) were purchased from Sinopharm Chemical Reagent Co., Ltd. The sample was extracted with benzene-ethanol (2∶1, v/v) in a Soxhlet for 4 h, and the remaining pellet was collected as cell wall residue (CWR). The procedure of nitrobenzene oxidation of lignin was conducted as follows; 0.05 g CWR was added with 5 mL 2 M NaOH and 0.5 mL nitrobenzene, and a stir bar was put into a 25 mL Teflon gasket in a stainless steel bomb. The bomb was sealed tightly and heated at 170°C (oil bath) for 3.5 h and stirred at 20 rpm. Then, the bomb was cooled with cold water. The chromatographic internal standard (ethyl vanillin) was added to the oxidation mixture. This alkaline oxidation mixture was washed 3 times with 30 mL CH_2_Cl_2_/ethyl acetate mixture (1∶1, v/v) to remove nitrobenzene and its reduction by-products. The alkaline solution was acidified to pH 3.0–4.0 with 6 M HCl, and then extracted with CH_2_CI_2_/ethyl acetate (3×30 mL) to obtain the lignin oxidation products which were in the organic phase. The organic extracts were evaporated to dryness under reduced pressure at 40°C. The oxidation products were dissolved in 10 mL chromatographic pure methanol.

For HPLC analysis the solution was filtered with a membrane filter (0.22 µm). Then 20 µL solution was injected into the HPLC (Waters 1525) column Kromat Universil C18 (4.6 mm×250 mm, 5 µm) operating at 28°C with CH_3_OH:H_2_O:HAc (16∶63∶1, v/v/v) carrier liquid (flow rate: 1.1 mL/min). Calibration curves routinely yielded correlation coefficients 0.999 or better, and the components were detected with a UV-detector at 280 nm.

### Measurement of lignocellulose crystallinity

The X-ray diffraction (XRD) method was applied for detection of cellulose crystallinity index (CrI) as described by Zhang et al. [7] and Wu et al. [8]. The biomass samples were examined by means of wide-angle X-ray diffraction on a Rigaku-D/MAX instrument (Uitima III, Japan) with 0.0197°/s from 10° to 45°. The crystallinity index (CrI) was estimated using the intensity of the 200 peak (I_200_, θ = 22.5°) and intensity at the minimum between the 200 and 110 peaks (I_am_, θ = 18.5°), based on the equation: CrI = 100×(I_200_-I_am_)/I_200_. I_200_ represents both crystalline and amorphous materials while I_am_ represents amorphous material. The standard error of the CrI method was detected at ±0.05 to approximately 0.15 using five representative samples in triplicates.

### Scanning electron microscopy (SEM) observations

Scanning electron microscopy (SEM) was used to examine the biomass residue, as described by Li et al. [6]. The well-mixed biomass powder samples were pretreated with 1% NaOH or 1% H_2_SO_4_, and hydrolyzed with the mixed-cellulases. Then, the lignocellulose samples were rinsed with distilled water until the pH was 7.0, dried under air, and sputter-coated with gold in a JFC-1600 ion sputter (Mito City, Japan). The surface morphology of the treated samples was observed by SEM (JSM-6390/LV, Hitachi, Tokyo, Japan), and the representative imagines of each sample were photographed from 5–10 views.

### Chemical pretreatment of biomass samples

The biomass pretreatments were performed as previously described by Huang et al. [27], Li et al. [6] and Wu et al. [8] with minor modifications. All samples were carried out in triplicate. H_2_SO_4_ pretreatment: the well-mixed powder of biomass sample (0.3 g) was added with 6 mL H_2_SO_4_ at three concentrations (0.25%, 1%, 4%, v/v). The tube was sealed and heated at 121°C for 20 min in an autoclave (15 psi) after sample was mixed well. Then, the tube was shaken at 150 rpm for 2 h at 50°C, and centrifuged at 3,000 *g* for 5 min. The pellet was washed three times with 10 mL distilled water, and stored at −20°C for enzymatic hydrolysis. All supernatants were collected for determination of total sugars (pentoses and hexoses) released from acid pretreatment, and samples with 6 mL distilled water were shaken for 2 h at 50°C as the control.

NaOH pretreatment: the well-mixed powder of biomass sample (0.3 g) was added with 6 mL NaOH at three concentrations (0.5%, 1%, 4%, w/v). The tube was shaken at 150 rpm for 2 h at 50°C, and centrifuged at 3,000 *g* for 5 min. The pellet was washed three times with 10 mL distilled water, and stored at −20°C for enzymatic hydrolysis. All supernatants were collected for determination of total sugars released from alkali pretreatment, and samples with 6 mL distilled water were shaken for 2 h at 50°C as the control.

### Enzymatic hydrolysis of lignocellulose residues

The lignocellulose residues obtained from various pretreatments were washed 2 times with 10 mL distilled water, and once with 10 mL mixed-cellulase reaction buffer (0.2 M acetic acid-sodium acetate, pH 4.8). The washed residues were added with 6 mL (1.6 g/L) of mixed-cellulases containing β-glucanase (≥2.98×10^4^ U), cellulase (≥298 U) and xylanase (≥4.8×10^4^ U) from Imperial Jade Bio-technology Co., Ltd). During the enzymatic hydrolysis, the samples were shaken under 150 rpm at 50°C for 48 h. After centrifugation at 3,000 *g* for 10 min, the supernatants were collected for determining amounts of pentoses and hexoses released from enzymatic hydrolysis. The samples with 6 mL reaction buffer were shaken for 48 h at 50°C as the control. All samples were carried out in triplicate.

### Statistical calculation of correlation coefficients

The statistical software (SPSS 17.0) was applied for any statistical analysis. Correlation coefficient values were calculated by performing Spearman rank correlation analysis for all pairs of the measured aspects (or traits, factors) across the whole populations. The measured aspects were derived from the average values of duplications. The box plot, histogram and line graph presented in the study were generated by using software (Origin 8.0).

## Results and Discussion

### Effects of major wall polymers on biomass digestibility in corn

**Figure 1:**
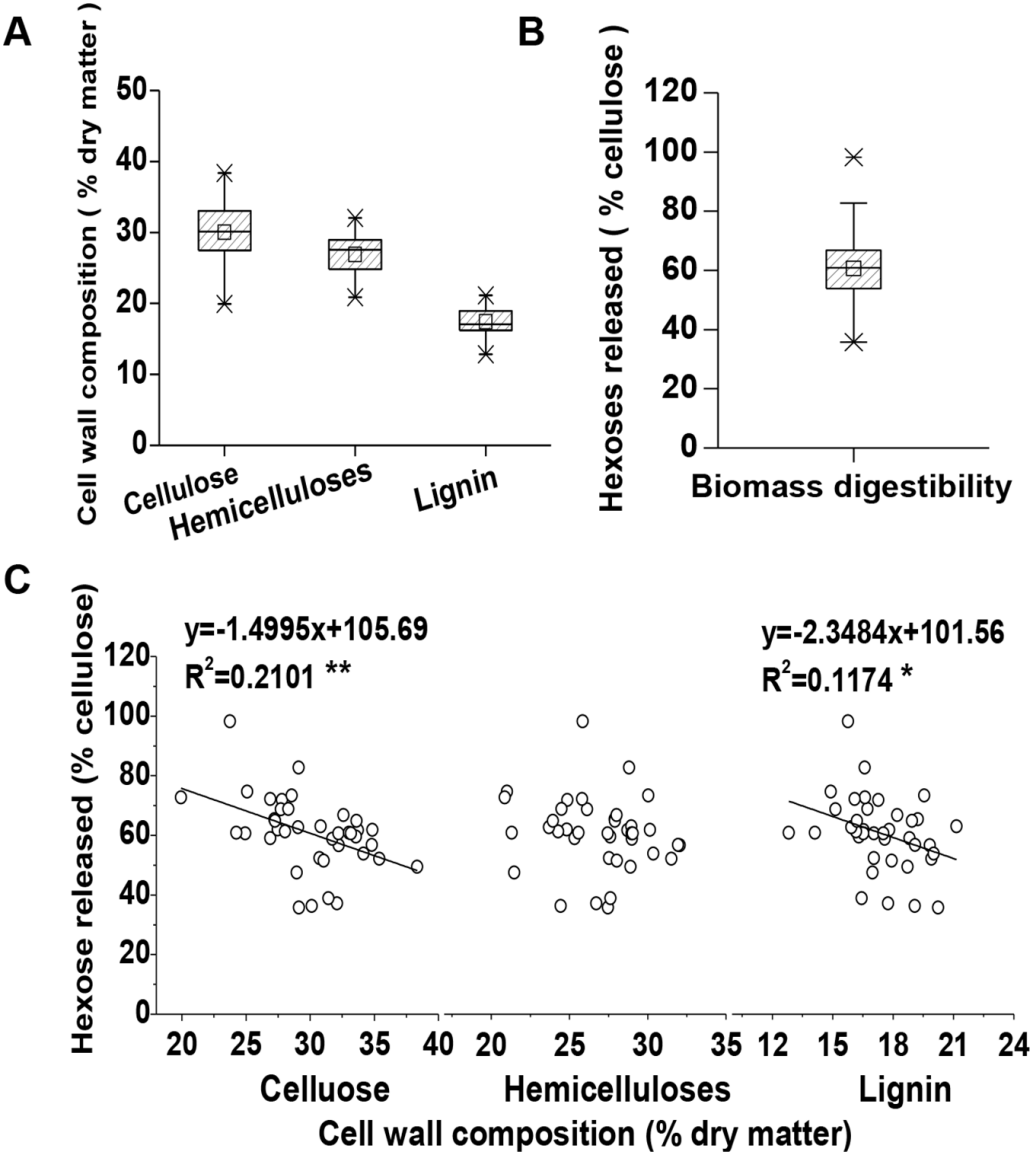
Effects of major wall polymer levels on biomass digestibility in total 40 representative corn accessions. (**A**) Variations of three major wall polymers (cellulose, hemicelluloses and lignin); (**B**) Diversity of hexoses yields from enzymatic hydrolysis after 1% NaOH pretreatment; (**C**) Correlations between wall polymers and hexoses yields after 1% NaOH pretreatment. * and ** Indicated as significant correlations at *p*<0.05 and 0.01 (n = 40), respectively.

Corn is a typical C4 food crop with large amounts of lignocellulose residues. Although corn stove has been applied in biofuels, little information is available about the characteristics of wall polymer involved in biomass process [8], [12]. In the current study, a total of 40 corn samples were selected from hundreds of natural corn accessions collected worldwide, including various ecological types and genetic germplasms (Fig. 1). In general, the selected corn samples exhibited a diverse cell wall composition (Fig. 1A, Table S1). For instance, the cellulose contents vary from 19.94% to 38.35% (% dry matter), hemicelluloses from 20.89% to 32.04%, and lignin from 12.83% to 21.16%. Hence, the corn samples showed a relatively low average lignin level compared with *Miscanthus* [5] and wheat [8].

Biomass enzymatic digestibility (saccharification) has been defined by measuring the hexoses yields (% cellulose) released by hydrolysis of lignocellulose using crude cellulase mixture after the samples were exposed to various pretreatment conditions [5], [6], [8]. The hexoses yields of the 40 corn samples were measured after they were pretreated with 1% NaOH (Fig. 1B, Table S1). As a result, the corn samples displayed diverse biomass digestibility with hexoses yields ranging from 35.76% to 98.16%, and almost half of the samples exhibited high hexoses yields up to 60%. Hence, the 40 corn samples were suitable for the experiments investigating the effects of wall polymers on biomass enzymatic digestibility.

Correlation analysis has been extensively performed to determine the effects of wall polymer on biomass saccharification in plants [7], [8], [27]. In this study, the correlations were analyzed between the three major wall polymer levels and hexoses yields after the 40 corn samples were pretreated with 1% NaOH (Fig. 1C). As a result, cellulose and lignin showed a significantly negative correlation with hexoses yields at *p*<0.05 and 0.0l levels. By comparison, hemicelluloses were not correlated with hexoses yields (*p*>0.05), different from the previous observations in *Miscanthus* [5], rice, and wheat [8], but similar to the finding in sweet sorghum [33]. Hence, the hemicelluloses level is not the main factor affecting biomass enzymatic saccharification in corn.

### Analysis of biomass digestions in five typical pairs of corn samples

**Figure 2:**
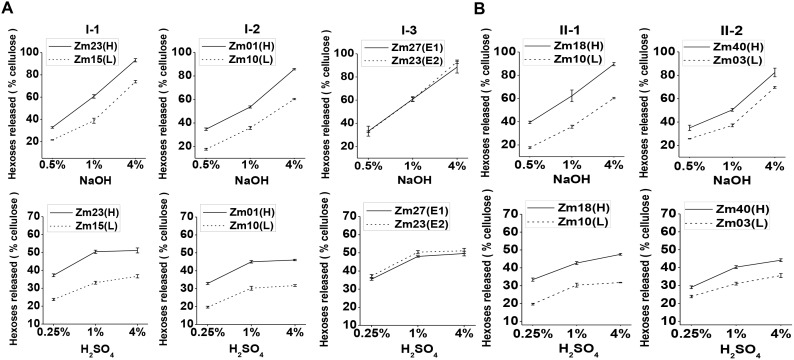
Hexoses yields released from enzymatic hydrolysis after NaOH and H_2_SO_4_ pretreatments in corn samples. **(A)** Pairs I-1, I-2 and I-3 samples; **(B)** Pairs II-1 and II-2 samples. Bar indicated as ± SD (n = 3).

To test the effects of major wall polymer levels on biomass digestibility, we selected five standard pairs of corn samples (Table 1), and compared their hexoses yields released from enzymatic hydrolysis after various chemical pretreatments (Fig. 2). Each of the three pairs (I-1, I-2, and I-3) displayed significant changes in single wall polymers (cellulose, lignin, and hemicelluloses; *p*<0.01) by 26.02%, 18.72%, and 28.80%, respectively. The two other major wall polymers of each pair did not show significant differences less than 6% (Table 1). Pretreated with three different concentrations (0.25% or 0.5%, 1%, and 4%) of NaOH and H_2_SO_4_, the Zm23 and Zm01 samples of pairs I-1 and I-2 with less cellulose and lignin contents (Table 1), respectively exhibited significantly 1.6- and 1.9-fold higher hexoses yields than those of their paired samples (Zm15 and Zm10; *p*<0.01; Fig. 2A, Table S2). The Zm27 sample of pair I-3 contained 28.80% less hemicelluloses showed the similar hexoses yields to its paired Zm23 sample from NaOH pretreatments or slightly lower hexoses yields from H_2_SO_4_ pretreatments. Hence, the cellulose and lignin levels exhibited significantly negative effects on hexoses yields from various chemical pretreatments, whereas the hemicelluloses did not affect biomass enzymatic digestibility in corn.

**Table 1:** Cell wall composition (% dry matter) of biomass residues in typical pairs of corn samples.

Pair	Sample	Cell wall composition (% dry matter)					
		Cellulose		Hemicelluloses		Lignin	
I-1	Zm23(H)^a^	24.94±0.43**	**–26.02%** b	27.46±0.43	–0.59%	16.52±0.33	0.50%
	Zm15(L)	**31.43±0.55**		27.62±0.30		16.44±1.00	
I-2	Zm01(H)	30.74±0.84	5.57%	27.54±0.55	0.27%	17.05±0.46**	**–18.72%**
	Zm10(L)	29.12±0.29		27.46±0.20		**20.25±0.14**	
I-3	Zm27(E1)	24.26±0.31	–2.82%	21.32±0.56**	**–28.80%**	16.42±0.85	–0.65%
	Zm23(E2)	24.94±0.43		**27.46±0.43**		16.52±0.33	
II-1	Zm18(H)	30.82±0.74	5.84%	28.96±0.69	5.46%	21.16±0.60	4.49%
	Zm10(L)	29.12±0.29		27.46±0.50		20.25±0.54	
II-2	Zm40(H)	31.05±0.59	–3.38%	28.03±0.45*	4.90%	17.93±0.46	0.96%
	Zm03(L)	32.10±0.61		26.72±0.07		17.76±0.29	

* and ** Indicated significant difference between the two samples of each pair by *t*-test at *p*<0.05 and 0.01, respectively (n = 3).

a Sample in the pair with high (H) or low (L) or equal (E) biomass digestibility.

b Percentage of the increased or decreased level between the two samples of each pair: subtraction of two samples divided by low value.

Furthermore, we detected other two typical pairs of corn sample (II-1, II-2) that each displayed a similar cell wall composition with wall polymer alterations by less than 6% (Table 1). Although the three major wall polymer levels of paired samples were slightly different, both pairs displayed remarkable changes in hexoses yields after these samples were pretreated with NaOH and H_2_SO_4_ (Fig. 2B, Table S2). In particular, pair II-1 exhibited the highest increase in hexoses yields, reaching a maximum of 2.2-fold increase after pretreated with 0.5% NaOH, which was even higher than that of Pair I-2 (1.9-fold increase). Thus, the data indicated that the wall polymer content should not be the major factor on biomass enzymatic digestibility, but the wall polymer features may play a dominant role as reported in wheat and rice species [8].

### Detection of wall polymer features in five pairs of samples

**Figure 3:**
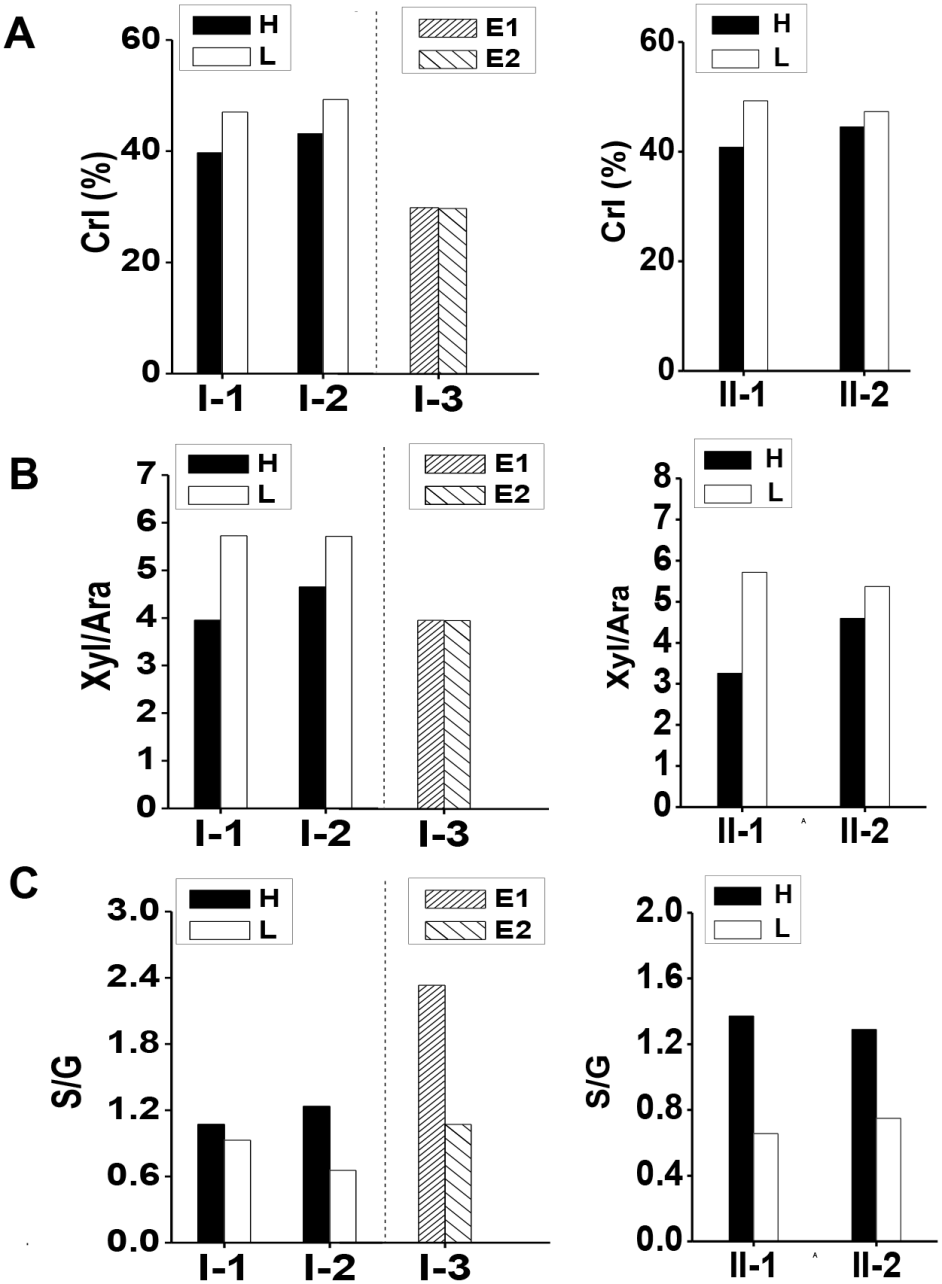
Detection of cell wall features in typical five pairs of corn samples. (**A**) Lignocellulosic CrI of raw material. (**B**) Xyl/Ara ratio of the non-KOH-extractable hemicelluloses; (**C**) S/G ratio of the non-KOH-extractable lignin. H/L/E Indicated as relatively high/low/equal biomass digestibility at pair.

The crystalline index (CrI) of lignocellulose has been used to determine cellulose crystallinity in plants [5], [7], [8]. In the present study, the CrI of lignocellulose in the five standard pairs of corn samples was examined (Fig. 3A, Table S3). Corn samples with relatively high biomass digestibility (Fig. 2) in the four sample pairs (I-1, I-2, II-1, and II-2) exhibited lower CrI values than that of their paired samples (Fig. 3A). The reduced CrI ratios ranged from 6% to 17% in the four pairs of corn samples (Table S3). By comparison, pair I-3 samples did not show different CrI values, consistent with their similar biomass digestibility (Fig. 2). Hence, the results indicated that lignocellulose CrI was also a key factor on biomass enzymatic saccahrification in corn.

With regard to the hemicelluloses feature, we determined monosaccharide composition of hemicelluloses in the five standard pairs of corn samples (Table S4). Similar to *Miscanthus*, rice, wheat and sweet sorghum [5], [8], [33], all corn samples contained two major pentoses: xylose (Xyl) and arabinose (Ara), indicating that xylan was one of major hemicelluloses. Significant amount glucose was also found in hemicelluloses, suggesting that a rich β-1,3; 1,4-glucans could also be present in corn [16], [30]. Considering that the substitution degree of Ara in xylan has been reported as a major factor affecting biomass enzymatic digestibility in grasses [6], [8], we calculated Xyl/Ara values as the reverse indicator of the substitution degree of Ara in xylan in two types of hemicelluloses (KOH-extractable and non-KOH-extractable). However, only the non-KOH-extractable hemicelluloses, neither the KOH-extractable nor total hemicelluloses, displayed consistently reduced Xyl/Ara ratios in the samples of four pairs (Pairs I-1, I-2, II-1, II-2) with relatively higher hexoses yields (Table S4 and Fig. 3B). By comparison, pair I-3 samples with similar hexoses yields (Fig. 2), showed close Xyl/Ara values in the non-KOH-extractable hemicelluloses. Hence, the substitution degree (reverse Xyl/Ara) of Ara in the non-KOH-extractable xylan was a positive factor affecting biomass saccharification in corn. However, corn is distinct from *Miscanthus* that displays the positive effects of the substitution degrees of Ara in both KOH-extractable and non-KOH-extractable xylans [6].

Lignin has been recently characterized with dual effects on biomass enzymatic digestibility due to monolignin constitution distinctive in different plant species [5], [8], [18], [19], [34]. In the present study, three monolignin ratios (S/G, H/G, and S/H) were calculated from KOH-extractable and non-KOH-extractable lignins in the five pairs of corn samples (Table S5). The biomass samples with relatively high hexoses yields exhibited the higher S/G ratios than that of their paired samples in the non-KOH-extractable lignin, rather than KOH-extractable or total lignin (Fig. 3C, Table S5). This result suggested that the S/G ratios in the non-KOH-extractable lignin could be applied as the positive indicators on biomass saccharification in corn. However, this result is contrary to that in a previous study, in which the S/G of *Miscanthus* was a negative indicator on biomass saccharification [5]. In addition, the corn was different from the rice and wheat that displayed a positive impact of H/G in the KOH-extractable lignin, rather than the non-KOH-extractable residue [8]. Although pair I-3 exhibited similar hexoses yields (Fig. 2A), two samples showed a different S/G in the non-KOH-extractable lignin (Fig. 3C).

### Observation of biomass residues from pretreatment and enzymatic hydrolysis

**Figure 4:**
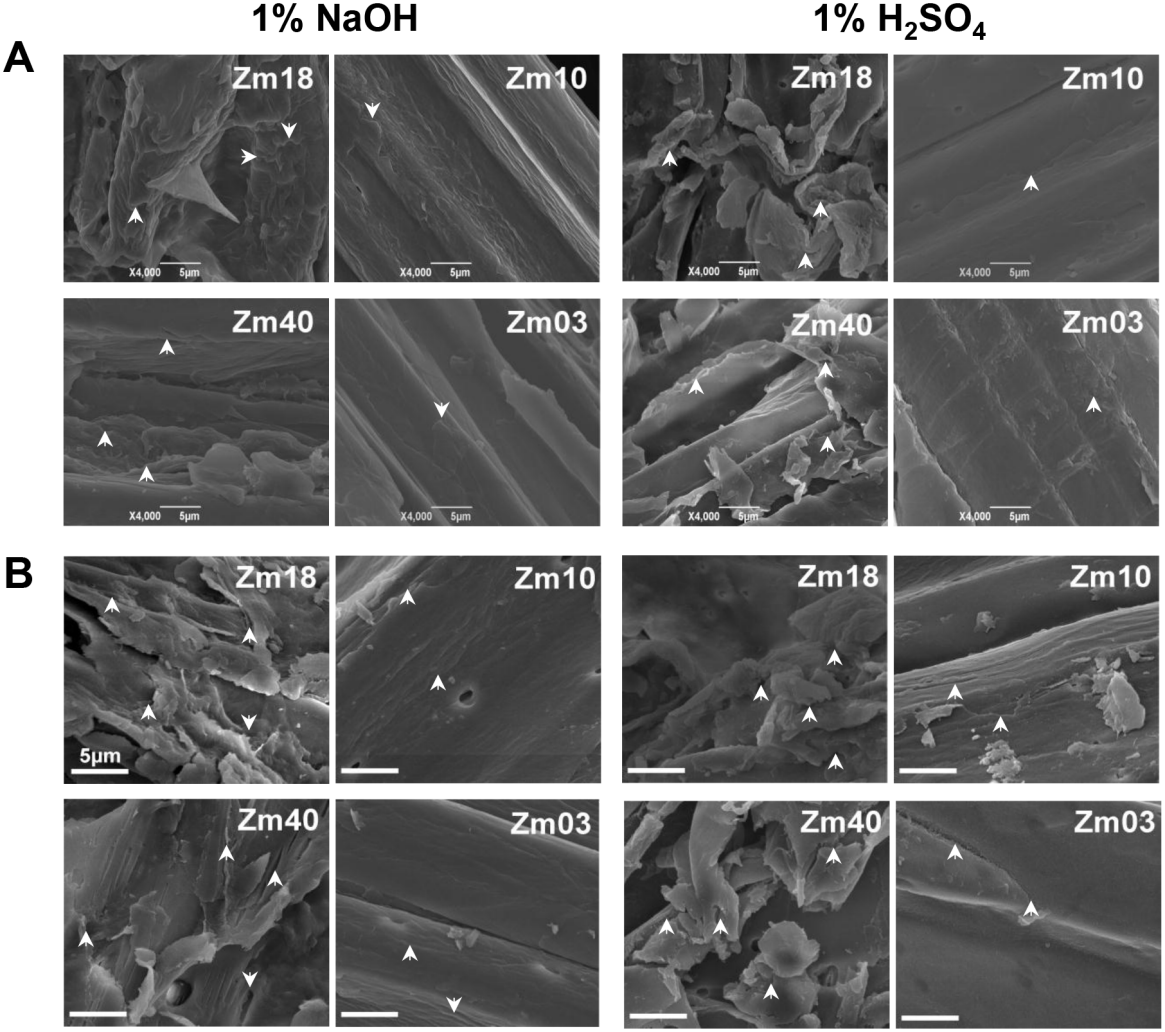
Scanning electron microscopic observation of biomass residues in pairs II-1 and II-2 corn samples. (**A**) Biomass residues released from pretreatments with 1% NaOH and 1% H_2_SO_4_; (**B**) Biomass residues released from enzymatic hydrolysis after 1% NaOH and 1% H_2_SO_4_ pretreatments. Arrow indicated the rough face.

Biomass residue surfaces in pairs II-1 and II-2 samples were observed under scanning electron microscopy (Fig. 4). After pretreated with 1% NaOH and 1% H_2_SO_4_, the Zm18 and Zm40 samples of pairs II-1 and II-2 with relatively high hexoses yields displayed coarse biomass residue surfaces, whereas their paired samples (Zm10 and Zm03) showed smooth surfaces (Fig. 4A). In sequential enzymatic hydrolysis, both Zm18 and Zm40 samples exhibited rougher surfaces than the pretreated samples (Fig. 4B); this result is similar to that in previous studies on *Miscanthus*, rice, and wheat [5], [7], [8]. Hence, the rough surface of the biomass residue could indicate a relatively effective biomass enzymatic hydrolysis. It could also suggest that biomass residue surface was mainly affected by the characteristics of wall polymers because pairs II-1 and II-2 showed similar cell wall compositions.

### Correlation among wall polymer features and biomass saccharification

**Figure 5:**
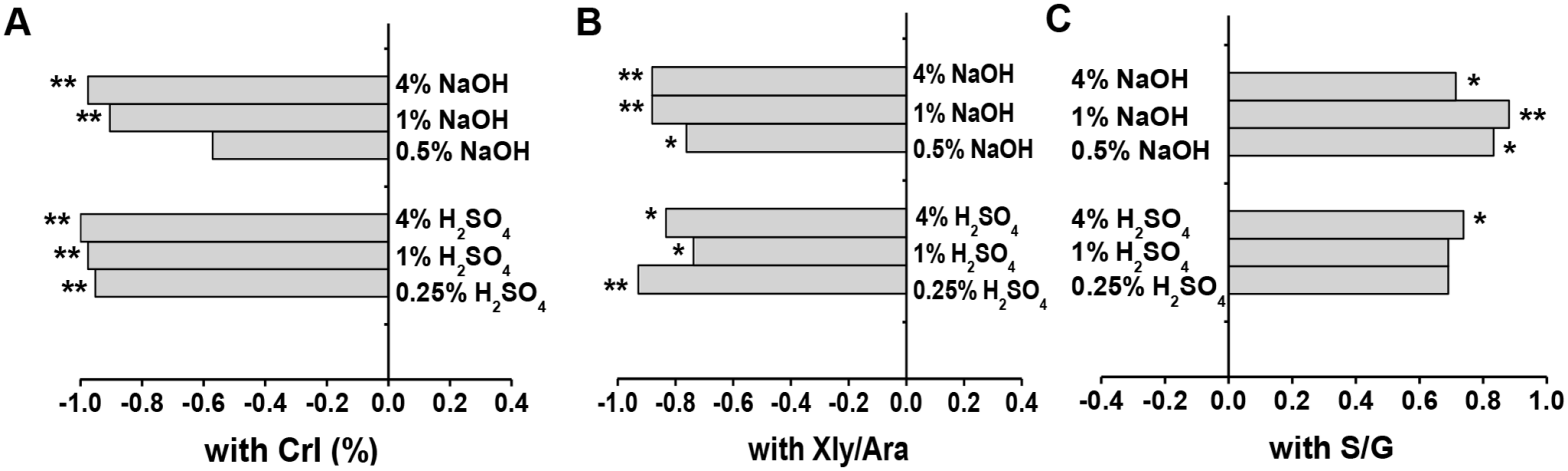
Correlation analysis between wall polymer features and hexoses yields from enzymatic hydrolysis after pretreatments. (**A**) Correlative coefficients between lignocellulose CrI (%) of raw material and the hexoses yields (% cellulose) released from enzymatic hydrolysis after NaOH and H_2_SO_4_ pretreatments at three concentrations; (**B**) Coefficients with Xyl/Ara of the non-KOH-extractable hemicelluloses; (**C**) Coefficients with S/G of the non-KOH-extractable lignin. * and ** Indicated as significant correlations at *p*<0.05 and 0.01 levels (n = 8), respectively.

To confirm the predominant effects of wall polymer features on biomass digestibility, we further performed a correlation analysis by using the five pairs of corn samples (Fig. 5). A negative correlation was found between lignocellulose CrI and hexoses yields released from enzymatic hydrolyses after the samples were pretreated with NaOH and H_2_SO_4_ except 0.5% NaOH pretreatment (*p*<0.01 or *p*<0.05; Fig. 5A, Table S6). A similar result was not observed in the samples pretreated with 0.5% NaOH possibly because of relatively low hexose yields that caused small variations among the five pairs of corn samples. However, our results showed that the CrI of lignocellulose negatively affected biomass enzymatic saccharification in corn.

Furthermore, correlations were calculated between hexoses yields and Xyl/Ara ratios in the two types of hemicelluloses (Fig. 5B, Table S7). Only non-KOH-extractable hemicelluloses showed significantly negative correlations (*p*<0.01 or *p*<0.05) compared with KOH-extractable or total hemicelluloses. This result indicated that the non-KOH-extractable hemicelluloses predominantly affected biomass enzymatic digestion in corn. A positive correlation was also found between hexoses yields and S/G ratios in the non-KOH-extractable lignin, compared with KOH-extractable or total lignin (Fig. 5C, Table S8). Although the two pretreatment conditions (0.25% and 1% H_2_SO_4_) did not show significant correlations, correlation coefficients remained high (Table S8). Therefore, the non-KOH-extractable lignocellulosic characteristics could predominantly affect biomass enzymatic digestibility in corn. Considering that the S/G of the total lignin negatively affects biomass enzymatic digestibility in *Miscanthus* and the H/G of KOH-extractable lignin positively affects biomass enzymatic digestibility in wheat and rice [6], [8], we found that corn was different from wheat and rice because its non-KOH-extractable S/G elicited a positive effect on biomass enzymatic hydrolysis.

### Mechanism on the wall polymer features that affect biomass enzymatic digestion

**Figure 6:**
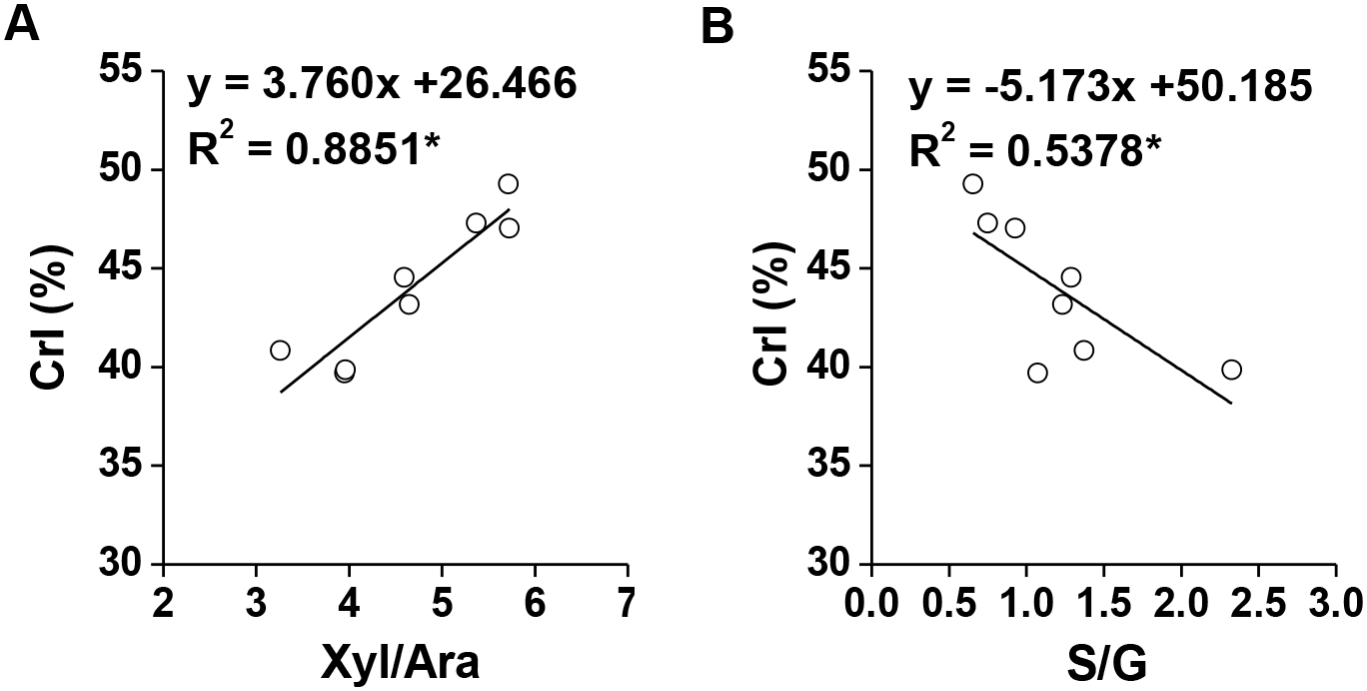
Correlation analysis between lignocellulose CrI and the non-KOH-extractable wall polymer features. (**A**) Xyl/Ara of non-KOH-extractable hemicelluloses; (**B**) S/G of the non-KOH-extractable lignin.*Indicated as significant correlations at *p*<0.05 levels (n = 8).

Lignocellulose crystallinity is the key factor that negatively affects biomass enzymatic digestibility in plants [5]–[8], [31], [32]. In general, lignocellulose crystallinity is distinctively affected by two major wall polymer (hemicelluloses and lignin) characteristics [5]–[8]. To understand the predominant effects of wall polymer characteristics on biomass enzymatic digestion in corn, we further performed a correlation analysis between lignocellulose CrI and two major wall polymer features (Xyl/Ara, S/G; Fig. 6). Our results showed that Xyl/Ara in non-KOH-extractable hemicelluloses exhibited a significantly positive correlation with lignocellulose CrI (*p*<0.05; R^2^ = 0.885; Fig. 6A, Table 2). Hence, the branched Ara of the non-KOH-extractable xylan may interact with β-1,4-glucan chains via hydrogen bonds that reduce the lignocellulose crystallinity for high biomass digestibility, as observed in other grasses [6]–[8]. By comparison, the non-KOH-extractable S/G was negatively correlated (*p*<0.05; R^2^ = 0.538; Fig. 6B, Table 2). This result suggested that G-monomer may be associated with β-1,4-glucan chains or S-monomer may interact with the Ara of xylan that indirectly reduces lignocellulose crystallinity. This result could also explain the different observation in pair I-3 regarding S/G, which did not affect hexoses yields, because the non-KOH-extractable S- and G-monomers may not affect lignocellulose crystallinity. However, the data confirmed that the non-KOH-extractable wall polymer features could play a predominant role in biomass enzymatic hydrolysis in corn.

**Table 2:** Correlative coefficients between lignocellulose CrI and two wall polymer features (Xyl/Ara, S/G).

	Xyl/Ara			S/G		
CrI (%)	KOH-extractable	Non-KOH-extractable	Total	KOH-extractable	Non-KOH-extractable	Total
	0.286	**0.833** *	0.595	–0.429	**0.738** *	–0.429

* Indicated significant difference at *p*<0.05 (n = 8).

### Potential cell wall modification for high biomass digestibility

Corn is the typical C4 food crop with enormous biomass residues for biofuels. However, desirable cell walls for high biomass digestibility can not be identified easily [1], because plant biomass is composed of many different cell types with diverse wall components. Furthermore, any genetic modification of plant cell walls could consequently lead to plant growth defect and mechanical strength reduction because of the cell wall is involved in diverse biological functions [12]. In the present study, we have screened out natural corn varieties with normal plant growth and high biomass digestibility. More importantly, we have found that the non-KOH-extractable Xyl/Ara predominately affect biomass enzymatic saccharification under various chemical pretreatments. Since the non-KOH-extractable biomass residue respectively covers 23.4% of total hemicelluloses and 12.0% of total lignin (Table 3), its genetic modification should cause less defects on plant growth and development than that of the KOH-extractable biomass. Despite that lignocellulose crystallinity is the key negative factor on biomass digestibility, it could be reduced by enhancing Ara substitution degree (reverse Xyl/Ara) in the non-KOH-extractable biomass. Hence, the current study indicated that mild cell wall modifications for enhancing biomass enzymatic saccharification could be performed by expressing genes involved in branched Ara biosynthesis or by partially silencing genes associated with xylan backbone synthesis in corn.

**Table 3:** Proportions of two types of hemicelluloses and lignin in the typical corn samples.

	KOH-extractable	Non-KOH-extractable	Total
Hemicelluloses	1341.4±122.6^a^	409.0±45.2	1750.4±140.1
	(1051.71496.5)^b^	(340.8477.4)	(1392.51874.1)
	76.60%^c^	23.40%	100%
Lignin	1064.4±100.3	144.7±45.5	1209.1±130.3
	(956.21273.5)	(86.4238.3)	(1093.71444.3)
	88.00%	12.00%	100%

a Mean value ± SD (n = 8);

b Minimum and maximum values;

c Percentage of total polymer.

## Conclusion

Correlative analysis of 40 representative corn germplasm accessions and comparative analysis of the five standard pairs of corn samples have demonstrated that either the substitution degree of Ara in xylan or the S/G of lignin in non-KOH-extractable biomass could positively affect biomass enzymatic digestibility under various chemical pretreatments by negatively affecting lignocellulose crystallinity. The results have provided the potential approaches that could be performed to modify plant cell wall for high biofuel production in corn.

## Supporting Information

Table S1 **Variations of wall polymers and biomass digestibility in total 40 corn accessions.**
(DOC)

Table S2 **Hexoses yields (% cellulose) released from enzymatic hydrolysis after NaOH and H_2_SO_4_ pretreatments in five typical pairs of corn samples.**
(DOC)

Table S3 **Lignocellulose crystaline index (CrI) of raw materials in the five typical pairs of corn samples.**
(DOC)

Table S4 **Monosaccharide composition of hemicelluloses.**
(DOC)

Table S5 **Monomer composition of lignin.**
(DOC)

Table S6 **Correlation coefficients between lignocellulose CrI values and hexoses yields from enzymatic hydrolysis after various chemical pretreatments in the typical corn samples.**
(DOC)

Table S7 **Correlation coefficients between hemicellulosic Xyl/Ara ratios and hexoses yields from enzymatic hydrolysis after various chemical pretreatments in the typical corn samples.**
(DOC)

Table S8 **Correlation coefficients between monolignin ratios and hexoses yields from enzymatic hydrolysis after various chemical pretreatments in the typical corn samples.**
(DOC)
