# Supplementary material for: Biomass Enzymatic Saccharification Is Determined by the Non-KOH-Extractable Wall Polymer Features That Predominately Affect Cellulose Crystallinity in Corn
Source: PLoS One. 2014 Sep 24;9(9):e108449. doi: 10.1371/journal.pone.0108449 (PMC4177209; doi:10.1371/journal.pone.0108449)
Supplement: Table S1 — Variations of wall polymers and biomass digestibility in total 40 corn accessions. (DOC) [file pone.0108449.s001.doc]

**Table S1. Variations of wall polymers and biomass digestibility in total 40 corn accessions.**

| Cell wall composition ( % dry matter ) | | | |  | Biomass digestibility |
| --- | --- | --- | --- | --- | --- |
| Cellulose | | Hemicelluloses | Lignin |  | Hexoses released (% cellulose) |
| 30.01±3.76**^＃^** | | 26.89±2.92 | 17.40±1.79 |  | 60.69±12.30 |
| (19.94～38.35)**^＆^** | (20.89～32.04) | | (12.83～21.16) |  | (35.76～98.16) |

**^＃^** Mean value ± SD (n = 40),**^＆^** Minimum and maximum values.
