# Supplementary material for: Biomass Enzymatic Saccharification Is Determined by the Non-KOH-Extractable Wall Polymer Features That Predominately Affect Cellulose Crystallinity in Corn
Source: PLoS One. 2014 Sep 24;9(9):e108449. doi: 10.1371/journal.pone.0108449 (PMC4177209; doi:10.1371/journal.pone.0108449)
Supplement: Table S2 — Hexoses yields (% cellulose) released from enzymatic hydrolysis after NaOH and H2SO4 pretreatments in five typical pairs of corn samples. (DOC) [file pone.0108449.s002.doc]

**Table S2. Hexoses yields (% cellulose) released from enzymatic hydrolysis after NaOH and H_2_SO_4_ pretreatments in typical pairs of corn samples.**

| Pair | Sample | NaOH | | | | |  |  | H_2_SO_4_ | | | | | |
| --- | --- | --- | --- | --- | --- | --- | --- | --- | --- | --- | --- | --- | --- | --- |
|  |  | 0.50% |  | 1% |  | 4% |  |  | 0.25% |  | 1% |  | 4% |  |
| I-1 | Zm23(H)**^a^** | **32.8±0.7**** | **1.5^b^** | **60.7±1.6**** | **1.6** | **93.2±1.5**** | **1.3** |  | **37.2±0.8**** | **1.6** | **50.4±0.9**** | **1.5** | **51.1±1.4**** | **1.4** |
|  | Zm15(L) | **21.6±0.2** |  | **38.8±2.3** |  | **73.9±1.1** |  |  | **23.7±0.6** |  | **33.1±0.8** |  | **36.7±0.9** |  |
| I-2 | Zm01(H) | **33.9±1.1**** | **1.9** | **52.4±0.9**** | **1.5** | **83.8±0.6**** | **1.4** |  | **32.9±0.6**** | **1.7** | **45.0±0.7**** | **1.5** | **46.0±0.4**** | **1.5** |
|  | Zm10(L) | **17.7±0.8** |  | **35.8±1.3** |  | **60.4±0.6** |  |  | **19.7±0.5** |  | **30.3±1.1** |  | **31.7±0.5** |  |
| I-3 | Zm27(E1) | 33.3±4.3 | 1.0 | 60.9±2.1 | 1.0 | 88.6±5.1 | 1.0 |  | **35.5±0.6*** | **0.95** | **48.0±0.4*** | **0.95** | 49.6±1.3 | 0.97 |
|  | Zm23(E2) | 32.8±0.7 |  | 60.7±1.6 |  | 93.2±1.5 |  |  | **37.2±0.8** |  | **50.4±0.9** |  | 51.1±1.4 |  |
|  |  |  |  |  |  |  |  |  |  |  |  |  |  |  |
| II-1 | Zm18(H) | **39.4±1.1**** | **2.2** | **63.0±5.0**** | **1.8** | **89.5±1.4**** | **1.5** |  | **33.4±0.9**** | **1.7** | **42.7±0.8**** | **1.4** | **47.5±0.5**** | **1.5** |
|  | Zm10(L) | **17.7±0.8** |  | **35.8±1.3** |  | **60.4±0.6** |  |  | **19.7±0.5** |  | **30.3±1.1** |  | **31.7±0.5** |  |
| II-2 | Zm40(H) | **35.0±2.3*** | **1.4** | **50.4±1.2**** | **1.4** | **82.4±3.7*** | **1.2** |  | **29.0±0.8**** | **1.2** | **40.3±0.8**** | **1.3** | **44.1±0.8**** | **1.2** |
|  | Zm03(L) | **25.8±0.2** |  | **37.2±1.2** |  | **69.7±0.8** |  |  | **23.9±0.7** |  | **31.1±0.8** |  | **35.6±1.1** |  |

***** and ****** Significant difference between the two samples of each pair by *t*-test at *p* < 0.05 and 0.01, respectively (n=3);**^a^** Sample at pair with relatively high (H) or low (L) or equal (E) biomass digestibility;**^b^** Ratio of two sample values at pair.
