# Supplementary material for: Biomass Enzymatic Saccharification Is Determined by the Non-KOH-Extractable Wall Polymer Features That Predominately Affect Cellulose Crystallinity in Corn
Source: PLoS One. 2014 Sep 24;9(9):e108449. doi: 10.1371/journal.pone.0108449 (PMC4177209; doi:10.1371/journal.pone.0108449)
Supplement: Table S3 — Lignocellulose crystaline index (CrI) of raw materials in the five typical pairs of corn samples. (DOC) [file pone.0108449.s003.doc]

**Table S3.** **Lignocellulose crystaline index (CrI) of raw materials in the five typical pairs of corn samples**

| Lignocellulose CrI (%) | | | | | | | | | | | | | | | | | | |
| --- | --- | --- | --- | --- | --- | --- | --- | --- | --- | --- | --- | --- | --- | --- | --- | --- | --- | --- |
| I-1 | |  |  | I-2 | | |  | I-3 | | |  | II-1 | | |  | II-2 | | |
| Zm23(H) **^b^** | 39.70 | -16% **^a^** |  | Zm01(H) | 43.16 | -12% |  | Zm27(E1) | 39.85 | 0.4% |  | Zm18(H) | 40.83 | -17% |  | Zm40(H) | 44.54 | -6% |
| Zm15(L) | 47.03 |  |  | Zm10(L) | 49.27 |  |  | Zm23(E2) | 39.70 |  |  | Zm10(L) | 49.27 |  |  | Zm03(L) | 47.29 |  |

**^a^** Percentage of the increased or decreased level between two samples of each pair: subtraction of two samples divided by low value;**^b^** Sample in the pair with relatively high (H) or low (L) or equal (E) biomass digestibility.
