# Supplementary material for: Biomass Enzymatic Saccharification Is Determined by the Non-KOH-Extractable Wall Polymer Features That Predominately Affect Cellulose Crystallinity in Corn
Source: PLoS One. 2014 Sep 24;9(9):e108449. doi: 10.1371/journal.pone.0108449 (PMC4177209; doi:10.1371/journal.pone.0108449)
Supplement: Table S4 — Monosaccharide composition of hemicelluloses. (DOC) [file pone.0108449.s004.doc]

**Table S4. Monosaccharide composition of hemicelluloses.**

| Pair | Sample | Total hemicelluloses composition (%) | | | | | | |  | Xyl/Ara | | | | | | | |
| --- | --- | --- | --- | --- | --- | --- | --- | --- | --- | --- | --- | --- | --- | --- | --- | --- | --- |
|  |  | Rha | Fuc | Ara | Xyl | Man | Gal | Glu |  | KOH-  extractable | |  | Non-KOH-  extractable | |  | Total | |
| I-1 | Zm23(H)**^b^** | 0.40% | ND**^c^** | 11.30% | 83.70% | 0.30% | 2.30% | 2.00% |  | 9.94 | -79.19%**^a^** |  | 3.95 | -44.96% |  | 7.43 | -63.38% |
|  | Zm15(L) | 0.30% |  | 7.30% | 89.20% | 0.20% | 1.70% | 1.30% |  | 17.82 |  |  | 5.73 |  |  | 12.14 |  |
|  |  |  |  |  |  |  |  |  |  |  |  |  |  |  |  |  |  |
| I-2 | Zm01(H) | 0.30% | ND | 8.90% | 86.70% | 0.30% | 1.80% | 2.00% |  | 12.89 | 7.40% |  | 4.65 | -22.94% |  | 9.75 | 2.81% |
|  | Zm10(L) | 0.30% |  | 9.20% | 87.00% | 0.20% | 2.00% | 1.30% |  | 12.01 |  |  | 5.71 |  |  | 9.48 |  |
| I-3 | Zm27(E1) | 0.30% | ND | 9.40% | 86.80% | 0.20% | 1.80% | 1.40% |  | 14.19 | 42.68% |  | 3.96 | 0.21% |  | 9.24 | 24.37% |
|  | Zm23(E2) | 0.40% |  | 11.30% | 83.70% | 0.30% | 2.30% | 2.00% |  | 9.94 |  |  | 3.95 |  |  | 7.43 |  |
|  |  |  |  |  |  |  |  |  |  |  |  |  |  |  |  |  |  |
| II-1 | Zm18(H) | 0.30% | ND | 10.80% | 83.80% | 0.40% | 2.60% | 2.10% |  | 10.57 | -13.57% |  | 3.26 | -75.41% |  | 7.74 | -22.54% |
|  | Zm10(L) | 0.30% |  | 9.20% | 87.00% | 0.20% | 2.00% | 1.30% |  | 12.01 |  |  | 5.71 |  |  | 9.48 |  |
|  |  |  |  |  |  |  |  |  |  |  |  |  |  |  |  |  |  |
| II-2 | Zm40(H) | 0.20% | ND | 8.30% | 87.80% | 0.20% | 1.70% | 1.70% |  | 15.31 | 26.86% |  | 4.59 | -16.87% |  | 10.58 | 9.57% |
|  | Zm03(L) | 0.20% |  | 9.00% | 86.90% | 0.30% | 1.80% | 1.80% |  | 12.07 |  |  | 5.37 |  |  | 9.66 |  |

**^a^** Percentage of the increased or decreased level between the two samples of each pair: subtraction of two samples divided by low value;**^b^** Sample in the pair with relatively high (H) or low (L) or equal (E) biomass digestibility;**^C^** ND – not detected.
