# Supplementary material for: Biomass Enzymatic Saccharification Is Determined by the Non-KOH-Extractable Wall Polymer Features That Predominately Affect Cellulose Crystallinity in Corn
Source: PLoS One. 2014 Sep 24;9(9):e108449. doi: 10.1371/journal.pone.0108449 (PMC4177209; doi:10.1371/journal.pone.0108449)
Supplement: Table S5 — Monomer composition of lignin. (DOC) [file pone.0108449.s005.doc]

**Table S5. Monomer composition of lignin.**

| Pair | Sample | Total lignin composition | | |  | KOH-extractable | | | | | |  | Non-KOH-extractable | | | | | | Total | | | | | | |
| --- | --- | --- | --- | --- | --- | --- | --- | --- | --- | --- | --- | --- | --- | --- | --- | --- | --- | --- | --- | --- | --- | --- | --- | --- | --- |
|  |  | H | G | S |  | S/G |  | H/G |  | S/H |  |  | S/G |  | H/G |  | S/H |  |  | S/G |  | H/G |  | S/H |  |
| I-1 | Zm23(H) **^b^** | 31.5% | 40.6% | 27.9% |  | 0.63 | -30%**^a^** | 0.87 | -100% | 0.72 | 54% |  | 1.07 | 15% | 0.18 | -230% | 6.06 | 280% |  | 0.69 | -21% | 0.78 | -100% | 0.89 | 66% |
|  | Zm15(L) | 45.9% | 29.5% | 24.6% |  | 0.81 |  | 1.74 |  | 0.47 |  |  | 0.93 |  | 0.58 |  | 1.59 |  |  | 0.83 |  | 1.55 |  | 0.54 |  |
| I-2 | Zm01(H) | 36.2% | 35.6% | 28.3% |  | 0.74 | 47% | 1.07 | -5% | 0.69 | 55% |  | 1.23 | 88% | 0.53 | 57% | 2.34 | 20% |  | 0.79 | 48% | 1.02 | 6% | 0.78 | 39% |
|  | Zm10(L) | 38.3% | 40.1% | 21.6% |  | 0.51 |  | 1.13 |  | 0.45 |  |  | 0.66 |  | 0.34 |  | 1.94 |  |  | 0.54 |  | 0.96 |  | 0.56 |  |
| I-3 | Zm27(E1) | 44.0% | 25.2% | 30.8% |  | 1.13 | 80% | 1.82 | 108% | 0.62 | -16% |  | 2.33 | 117% | 0.84 | 377% | 2.76 | -120% |  | 1.22 | 77% | 1.75 | 125% | 0.70 | -27% |
|  | Zm23(E2) | 31.5% | 40.6% | 27.9% |  | 0.63 |  | 0.87 |  | 0.72 |  |  | 1.07 |  | 0.18 |  | 6.06 |  |  | 0.69 |  | 0.78 |  | 0.89 |  |
|  |  |  |  |  |  |  |  |  |  |  |  |  |  |  |  |  |  |  |  |  |  |  |  |  |  |
|  |  |  |  |  |  |  |  |  |  |  |  |  |  |  |  |  |  |  |  |  |  |  |  |  |  |
| II-1 | Zm18(H) | 45.5% | 27.7% | 26.7% |  | 0.90 | 79% | 1.73 | 54% | 0.52 | 16% |  | 1.37 | 109% | 0.99 | 194% | 1.38 | -40% |  | 0.96 | 79% | 1.64 | 72% | 0.59 | 4% |
|  | Zm10(L) | 38.3% | 40.1% | 21.6% |  | 0.51 |  | 1.13 |  | 0.45 |  |  | 0.66 |  | 0.34 |  | 1.94 |  |  | 0.54 |  | 0.96 |  | 0.56 |  |
| II-2 | Zm40(H) | 37.1% | 32.7% | 30.2% |  | 0.87 | 25% | 1.25 | 18% | 0.70 | 7% |  | 1.29 | 72% | 0.29 | -25% | 4.42 | 115% |  | 0.92 | 31% | 1.13 | 19% | 0.81 | 10% |
|  | Zm03(L) | 35.8% | 37.7% | 26.5% |  | 0.69 |  | 1.06 |  | 0.65 |  |  | 0.75 |  | 0.36 |  | 2.05 |  |  | 0.70 |  | 0.95 |  | 0.74 |  |

**^a^** Percentage of the increased or decreased level between the two samples of each pair: subtraction of two samples divided by low value; ^b^ Sample in the pair with relatively high (H) or low (L) or equal (E) biomass digestibility.
