# Supplementary material for: Biomass Enzymatic Saccharification Is Determined by the Non-KOH-Extractable Wall Polymer Features That Predominately Affect Cellulose Crystallinity in Corn
Source: PLoS One. 2014 Sep 24;9(9):e108449. doi: 10.1371/journal.pone.0108449 (PMC4177209; doi:10.1371/journal.pone.0108449)
Supplement: Table S6 — Correlation coefficients between lignocellulose CrI values and hexoses yields from enzymatic hydrolysis after various chemical pretreatments in the typical corn samples. (DOC) [file pone.0108449.s006.doc]

**Table S6. Correlation coefficients between lignocellulose CrI values and hexoses yields from enzymatic hydrolysis after various chemical pretreatments in the typical corn samples.**

|  | NaOH | | |  | H_2_SO_4_ | | |
| --- | --- | --- | --- | --- | --- | --- | --- |
|  | 0.50% | 1% | 4% |  | 0.25% | 1% | 4% |
| CrI (%) | -0.571 | **-0.905**** | **-0.976**** |  | **-0.952**** | **-0.976**** | **-1.000**** |

****** Indicated significant difference at *p* < 0.01 level (n = 8).
