# Supplementary material for: Biomass Enzymatic Saccharification Is Determined by the Non-KOH-Extractable Wall Polymer Features That Predominately Affect Cellulose Crystallinity in Corn
Source: PLoS One. 2014 Sep 24;9(9):e108449. doi: 10.1371/journal.pone.0108449 (PMC4177209; doi:10.1371/journal.pone.0108449)
Supplement: Table S7 — Correlation coefficients between hemicellulosic Xyl/Ara ratios and hexoses yields from enzymatic hydrolysis after various chemical pretreatments in the typical corn samples. (DOC) [file pone.0108449.s007.doc]

**Table S7. Correlation coefficients between hemicellulosic Xyl/Ara ratios and hexoses yields from enzymatic hydrolysis after various chemical pretreatments in the typical corn samples.**

|  | Xyl/Ara | | | | |
| --- | --- | --- | --- | --- | --- |
|  | KOH-extractable |  | Non-KOH-extractable |  | Total |
| 0.5% NaOH | -0.048 |  | **-0.762 *** |  | -0.024 |
| 1% NaOH | -0.238 |  | **-0.881 **** |  | -0.071 |
| 4% NaOH | -0.381 |  | **-0.881 **** |  | -0.262 |
|  |  |  |  |  |  |
| 0.25% H_2_SO_4_ | -0.381 |  | **-0.881 **** |  | -0.190 |
| 1% H_2_SO_4_ | -0.214 |  | **-0.738 *** |  | -0.048 |
| 4% H_2_SO_4_ | -0.286 |  | **-0.833 *** |  | -0.119 |

***** and ****** Indicated significant difference at *p* < 0.05 and 0.01, respectively (n=8).
