# Supplementary material for: Biomass Enzymatic Saccharification Is Determined by the Non-KOH-Extractable Wall Polymer Features That Predominately Affect Cellulose Crystallinity in Corn
Source: PLoS One. 2014 Sep 24;9(9):e108449. doi: 10.1371/journal.pone.0108449 (PMC4177209; doi:10.1371/journal.pone.0108449)
Supplement: Table S8 — Correlation coefficients between monolignin ratios and hexoses yields from enzymatic hydrolysis after various chemical pretreatments in the typical corn samples. (DOC) [file pone.0108449.s008.doc]

**Table S8. Correlation coefficients between monolignin ratios and hexoses yields from enzymatic hydrolysis after various chemical pretreatments in the typical corn samples.**

|  | KOH-extractable | | |  | Non-KOH-extractable | | |  | Total lignin | | |
| --- | --- | --- | --- | --- | --- | --- | --- | --- | --- | --- | --- |
|  | S/G | H/G | S/H |  | S/G | H/G | S/H |  | S/G | H/G | S/H |
| 0.5% NaOH | 0.667 | 0.167 | 0.452 |  | **0.833 *** | 0.286 | 0.143 |  | 0.667 | 0.429 | 0.429 |
| 1% NaOH | 0.667 | 0.286 | 0.310 |  | **0.881 **** | 0.452 | 0.143 |  | 0.667 | 0.500 | 0.262 |
| 4% NaOH | 0.405 | 0.000 | 0.524 |  | **0.714 *** | 0.143 | 0.357 |  | 0.405 | 0.19 | 0.476 |
|  |  |  |  |  |  |  |  |  |  |  |  |
| 0.25% H_2_SO_4_ | 0.381 | -0.071 | 0.619 |  | 0.690 | 0.071 | 0.524 |  | 0.381 | 0.119 | 0.595 |
| 1% H_2_SO_4_ | 0.357 | -0.024 | 0.619 |  | 0.690 | 0.048 | 0.571 |  | 0.357 | 0.143 | 0.571 |
| 4% H_2_SO_4_ | 0.429 | 0.048 | 0.548 |  | **0.738 *** | 0.119 | 0.476 |  | 0.429 | 0.214 | 0.500 |

***** and ****** Indicated significant difference at *p* < 0.05 and 0.01, respectively (n=8).
